# Laparoscopic fixation of biological mesh at hiatus with glue and suture during hiatal hernia repair

**DOI:** 10.1186/s12893-021-01151-0

**Published:** 2021-03-22

**Authors:** Yusheng Nie, Yao Xiong, Lei Guan, Xin Yuan, Fuqiang Chen, Jie Chen, Huiqi Yang

**Affiliations:** Department of Hernia and Abdominal Wall Surgery, The Capital Medical University Beijing Chaoyang Hospital, Jingyuan Road No.5, Shijingshan District, Beijing, 100043 China

**Keywords:** Hiatal hernia, Biological mesh, Medical glue

## Abstract

**Background:**

Laparoscopic paraesophageal hernia repair is associated with higher recurrence rate. Mesh is used to reduce the recurrence rate. This retrospective study is to review our experience of biological mesh fixed with suture and medical glue in hiatal hernias repairs.

**Methods:**

A retrospective chart review was conducted for a consecutive series of patients undergoing laparoscopic hiatal herniorrhaphy between January 2018 and January 2019. After hiatus closure, a piece of biological prosthesis was fixed with medical glue and suture for reinforcement of the crural closure. Clinical outcomes were reviewed, and data were collected regarding operative details, complications, symptoms, and follow-up imaging. Radiological evidence of any size of hiatal hernia was considered to indicate a recurrence.

**Results:**

Thirty-six patients underwent surgery uneventfully without any serious complication. There was no mortality. The follow-up was, on average, 18.4 months, and there was no symptomatic recurrence. There was one anatomical recurrence without any related presentation. The method of mesh fixation with medical glue and suture took 12 min on average, and the handling was fairly easy.

**Conclusions:**

Biological mesh fixed with suture and medical glue was safe and effective for repairing large hiatal hernias. Of course, a longer follow-up is still needed for determining long-term outcomes.

## Background

Hiatal hernia is a common condition often associated with symptomatic gastroesophageal reflux disease (GERD) [1]. Laparoscopic hiatal hernia repair (LHHR) is now considered to be the gold standard for the management of hiatal hernias, and is associated with a reduced rate of perioperative morbidity and shorter hospital stay compared with the open approach [1, 2]. The standard steps include the excision of the sac, a thorough oesophageal mobilisation, primary closure of the hiatus, and a fundoplication [3, 4].

Oelschlager et al. reported that the recurrence rate after pure suture repair without mesh reinforcement is as high as 59% at 5-year follow-up [4]. Primary repair of the paraesophageal hiatal hernia is a significant risk factor for recurrence, especially when suturing the pillars of the diaphragm together under tension for the giant hiatal hernia. Two randomised trials have demonstrated that a significant reduction in recurrence rates can be achieved by using synthetic mesh for large hiatal hernia repair [5, 6]. However, a few synthetic mesh-related complications, such as mesh erosion, stricture and dysphagia, are reported [7, 8]. In order to avoid those complications, the surgical technique for reliable mesh fixation has been improved, and the mesh specially designed for hiatal hernia has also been improved.

On the other hand, recent studies have reported favourable results from using biological mesh for hiatal hernia repair [9, 10]. Due to the concern about potential complications related to synthetic mesh, biomaterial was adopted in the repair. However, the long-term results, especially for recurrence, still needs to be investigated further.

In our study, a retrospective study was conducted into laparoscopic hiatal hernia repair at our centre to review our experience with biological mesh for crural reinforcement and on mesh fixation with medical glue and suture.

## Methods

A retrospective study was conducted for a consecutive series of patients undergoing laparoscopic hiatal herniorrhaphy for symptomatic hiatal hernia between January 2018 and January 2019. Preoperative evaluation routinely included endoscopy, CT scan, upper gastrointestinal (UGI) series and oesophageal manometry test, and 24-h PH monitoring.

### Surgical technique

**Fig. 1:**
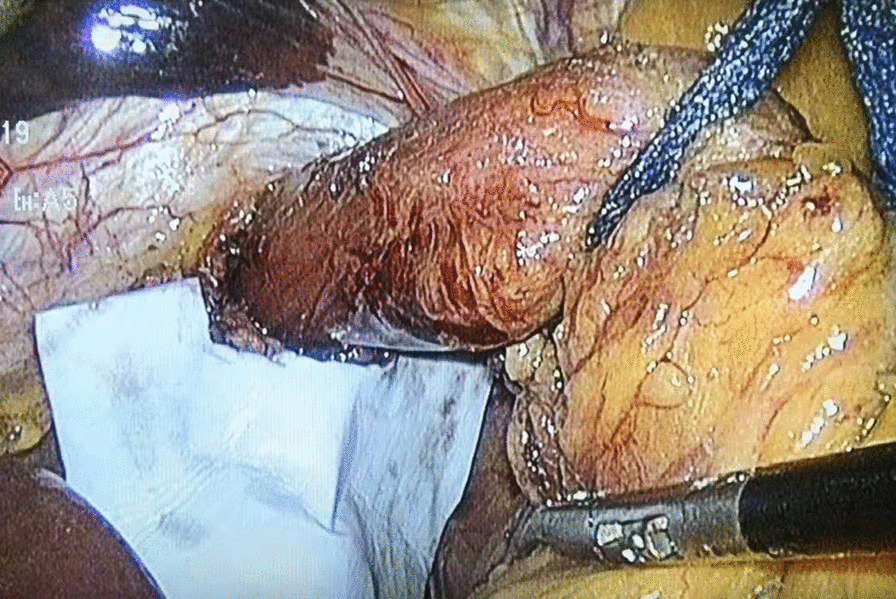
Initial placement of bioabsorbable mesh with medical glue (Compont Medical Adhesive) applied after cruroplasty

**Fig. 2:**
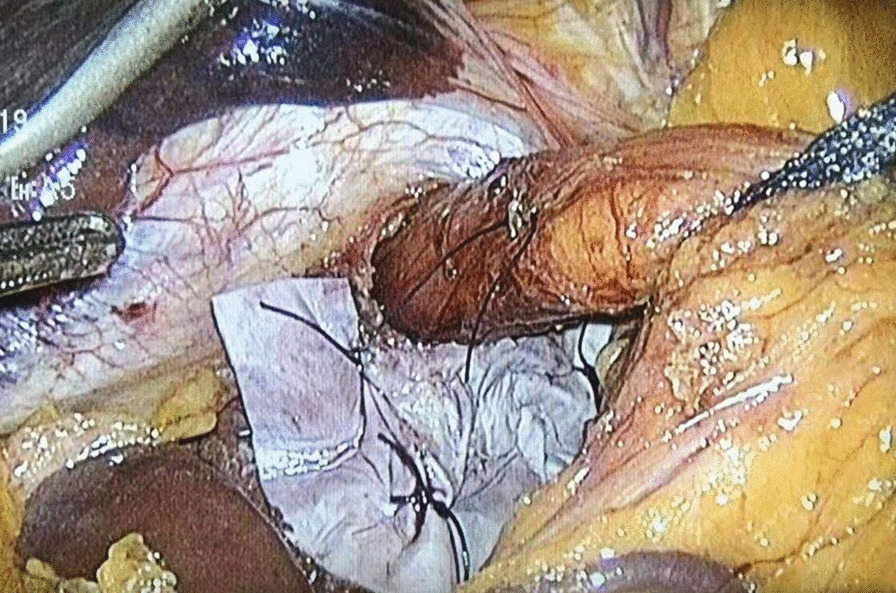
Three intermittent sutures further fix the prosthesis

One dose of antibiotics was administered at induction.Five laparoscopic ports were routinely used. Circumferential dissection of the hernia sac from the hiatus and mediastinal structures was performed. The sac was then first everted over the gastroesophageal junction and then excised. The hiatus was closed posteriorly with an interrupted suture (2-0 Prolene) to about 2.5 cm. A piece of biological prosthesis (ThomalGEN surgical patch, 6*8 cm, Guanhao Biotech Co. Ltd., Guangzhou, China) was prepared and cut in a U configuration, and was fixed with medical glue (Compont medical adhesive, 1.5ml/tube; Beijing Compont Medical Devices Co. Ltd., Beijing, China) for reinforcement of the crural closure (Fig. 1). The medical glue set consists of a sprayer, catheter, and n-butyl-2-cyanoacrylate (NBCA) glue. The catheter is introduced through one of the operating trocars and a 5mm grasper is inserted through the second trocar and used to direct the application of the glue. The mesh is fixed with four–five sprays. In addition, a three-point suture with three intermittent sutures (3−0 vicryl ) was used to further strengthen the prosthesis fixation (Fig. 2). Fundoplication was then performed with an interrupted suture (2-0 prolene) [11].

### Outcome measurement

Operative data were collected, including operation time (skin cut to closure), the time cost for prosthesis fixation, blood loss, any intraoperative complication, hiatus measurement, and the length of the intra-abdominal oesophagus after dissection.

Upper Gastrointestinal (UGI) Series: the preoperative and every 6-months postprocedural UGI series were performed at our medical centre. Preoperative exams were interpreted by the clinicians and were used as the primary means of hiatal hernia diagnosis. The radiologists were asked to formulate a consensus interpretation based on the following five-point scale: (1) Intact fundoplication located below the diaphragm without any portions of stomach seen above the plication; (2) Intact fundoplication, but with indeterminate positioning. Plication seen within 2 cm of the level of the left hemidiaphragm; (3) Intact plication with probable small sliding hiatal hernia. Plication seen between 2 cm and 5 cm above the left hemidiaphragm; (4) Intact plication with a large sliding hiatal hernia (5 cm above the left hemidiaphragm); (5) Slipped or disrupted fundoplication, and a portion of stomach present above the plication [9].

#### Symptom Questionnaire

A standardised symptom questionnaire was provided to patients two to four weeks after the operation, 6 months after the operation, and 1 year after the operation. Patients individually scored symptoms based on severity. The symptoms included in the questionnaire were: heartburn, chest pain, dysphagia, and gas bloating. The severity was scored using a visual analogue score (VAS), and patients marked each symptom from 0 to 10 (0 representing no effect on life and 10 representing extreme effect). In addition, the overall satisfaction with the outcome after surgery was scored from 0 to 10 (0 representing not satisfied and 10 representing highly satisfied) as well [11].

Endoscopy was routinely undertaken six months after the operation to evaluate if there was any recurrence.

SPSS 25.0 software was used for statistical analysis.

The protocol for this study was approved by the Clinical Research Ethics Committees at our hospital.

## Results

From January 2018 to January 2019, 36 patients underwent laparoscopic hiatal hernia repair with biological prosthesis fixed by medical glue and suture. Baseline Characteristics of patients is summarised in Table 1. There were 12 males (33.3%) and 24 females (66.7%) with a mean age of 68.4 ± 17.2 (range 31–84 years). The average BMI was 28.6 ± 6.8. No case underwent previous anti-reflux procedure.

**Table 1:** Baseline characteristics of patients

	Patients (n = 36)
Age (yr)	68.4 ± 17.2
Female	24 (66.7%)
BMI(kg/m^2^)	28.6 ± 6.8
Hypertension	15 (41.6%)
Diabetes	4 (11.1%)
Smoking status	9 (25%)
Chronic constipation	12 (33.3%)
COPD	3 (8.3%)
Hospital stay	4.2 ± 1.2

Preoperative oesophageal manometry showed that LESP on average was 6.8 ± 3.6mmHg, and 12 cases were found to have mild motility disorder. Endoscopy identified the hiatus hernia in all patients, and 7 cases had concomitant esophagitis.

The final diagnosis was confirmed during the operation. Two cases (5.6%) were sliding hiatal hernia (type I)with severe acid reflux, five cases (13.9 %) were giant hiatal hernia (type IV) containing at least two-thirds of the stomach, and 29 cases (80.5%) were paraesophageal hernia(type II and type III).

Anethesia evaluation included 31 cases of ASA grade 2 and 5 cases of ASA grade 3. All the patients underwent laparoscopic surgery uneventfully with an average operation time of 92.6 min (range 73–135 min). All surgery was performed by one experienced surgeon. The average blood loss was minimal (range 5–20ml). The width of hiatus on average was 4.4 ± 1.4 cm, and the length was 5.2 ± 1.2 cm. The length of intraabdominal esophagus was more than 3 cm. Fundoplication was performed in all cases. Intraoperative complications were described in four patients (11.1 %): three cases of pneumothorax (8.3%) during dissection of the sac that could be closed laparoscopically without placement of a chest tube, and one case of superficial liver laceration coagulated laparoscopically.

The mesh placement and fixation took approximately 12 min. The medical glue can provide a very solid fixation to the crural immediately, as the glue can be sprayed quite evenly in small particles. The suture fixation was completed by three intermittent sutures. The prosthesis was well secured, and there was no movement during the following fundoplication.

The analgesia was given when necessary. The patients started to mobilize 6 h after surgery.

**Fig. 3:**
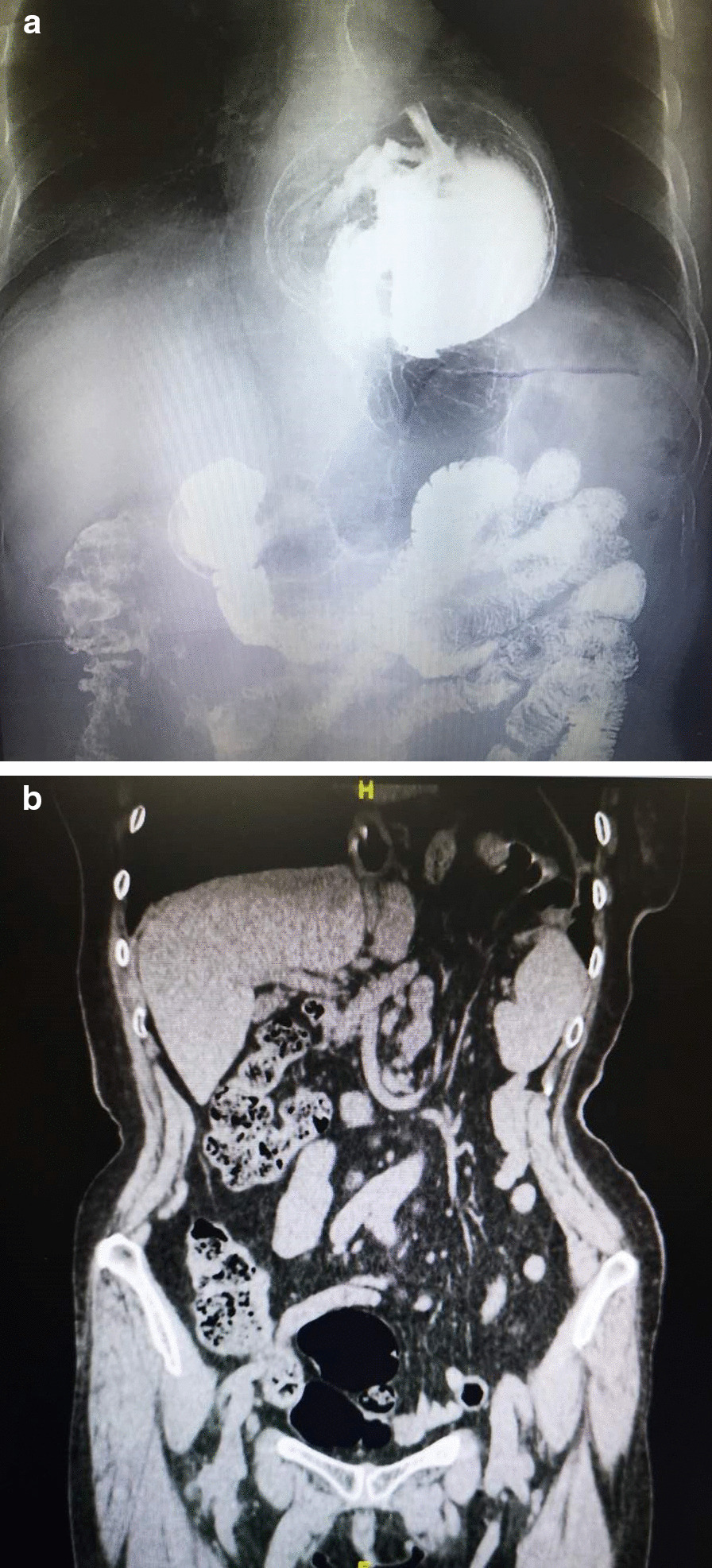
**a**, **b** Preoperative Upper GI study and CT scan showing a giant hiatal hernia

**Fig. 4:**
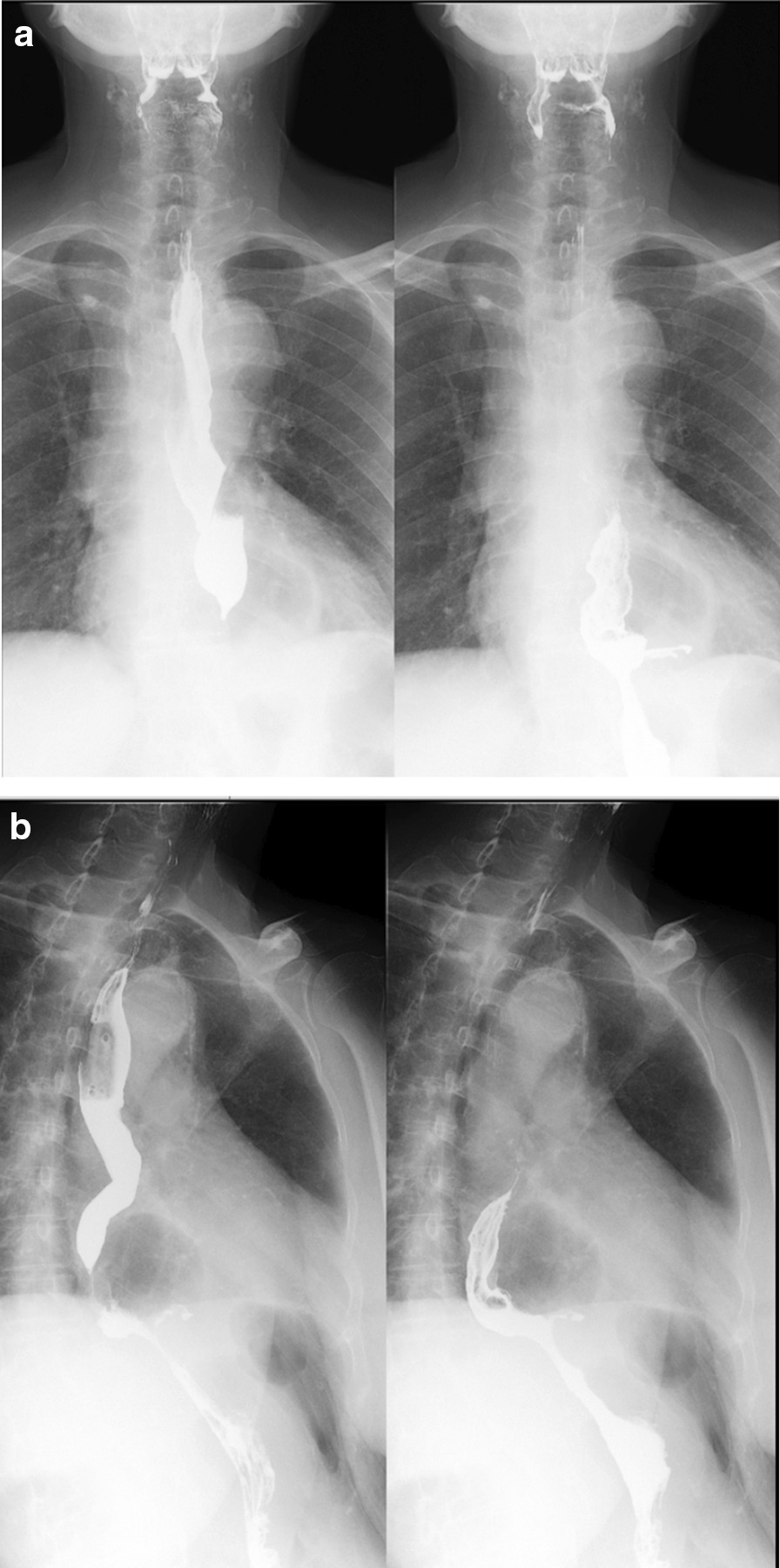
**a**, **b** Upper GI study at 1 year after the surgery showing sliding hiatal hernia

Clinical follow-up is summarised in Tables 2 and 3. The follow-up time was on average 18.4 months, ranging from 13 to 24 months. Overall, the clinical outcome was favourable. In general, most clinical symptoms such as heartburn,acid reflux, regurgitation, and chest pain improved significantly after the operation. The overall satisfaction score was 8 at 6 months follow up. Even though more than 50% experienced mild dysphagia and gas bloating after the operation, they improved gradually with diet instruction and all can eat normal diets at 6 months follow-up. No patient needed further intervention, such as endoscopic dilatation or reoperation. Endoscopy at 6 months follow-up, no recurrence was found. Among the 7 cases with concomitant esophagitis, 6 cases was cured, and one case was improved from Grade C to Grade A. At 1-year follow-up, one patient had asymptomatic recurrence shown at Upper GI series, and was scaled as 3 by a radiologist according to the above-mentioned criteria (Figs. 3 and 4).

**Table 2:** Preoperative and postoperative symptoms assessed using 0–10 visual analogue scale

Symptom	Preoperative	2–4 weeks	6ms
Heartburn	5.20 (3.92–6.47)	0.56 (0.13–1.00)*	0.48 (0.10–1.10)*
Chest pain	2.74 (1.58–3.88)	0.16 (0.02–0.38)*	0.28 (0.06–0.44)*
Regurgitation	3.62(1.84–4.32)	0.14(0.06–0.20)*	0.18(0.08–0.32)*
Acid reflux	5.70(3.82–7.80)	0.12(0.02–0.22)*	0.10(0.02–0.26)*
Dysphagia	/	0.57 (0.04–1.09)	0.14 (0.04–0.22)
Satisfactory score	/	8.36 (7.20–9.60)	8.18 (7.08–9.24)

All data are expressed as mean (95% CI’s); * P < 0.01 Compared to preoperative data

**Table 3:** Postoperative symptoms assessed using yes versus no questions

Symptom	2–4 weeks (%)	6ms
Heartburn	0	2.78%
Dysphagia	55.56	0
Bloating	77.78	5.56%
Diarrhoea	13.89	0

All data is % patients

There was no serious postoperative complication, and all were classified into Clavien- Dindo Grade 1. Only one case of asymptomatic recurrence did not need any treatment. No case needed further PPI treatment.

## Discussion

The application of bioabsorbable prosthesis such as Surgisis (small intestinal submucosa, SIS), BioA Tissue Reinforcement (polglycolide or teimethylene carbonate) or AlloDerm (human acellular dermis) in hiatal hernia repair has been suggested to avoid mesh related complications, such as erosion or stricture [9, 12, 13]. In our study, we used a prosthesis made of bovine pericardium for the reinforcement, and this mesh has been widely used in inguinal and ventral hernia repair in China. However, the concern on using biological prosthesis is the weaker strength compared to synthetic material, and this might result in higher recurrence. A few recent studies have confirmed reliable outcomes with biological prosthesis for hiatal hernia repair [13–15]. In our study, including more than 90% of giant or paraesophageal hernia, there was no symptomatic recurrence during follow-up. The case of sliding hernia shown at the Upper GI series 1 year after operation did not need further medical intervention. This patient was an 83-year-old female with a giant hiatal hernia, and we were advised to perform the surgery for a shorter time with lower CO2 pressure by the anaesthetist during the procedure for the safety. Therefore, a simple crural closure was performed without fundoplication, and this could be the reason why the fundus slipped upwards.

In this study, we used NCBA medical glue for prosthesis fixation after crural closure, and the results were satisfactory. The manipulation was easy, and fixation was secure. Previous animal studies have confirmed the safety of chemical and biological adhesives for mesh fixation [16–19]. Moreover, clinical studies have shown that adhesives, both biological and chemical, are an effective means of mesh fixation in hernia repair and results are comparable to those of traditional techniques such as suture and tack devices [20–27]. However, the shortcomings of biological sealants (e.g., fibrin glue), which are expensive, provide a weak bond, are slow to apply, and are potentially allergenic, have limited their application, particularly in China [7]. For these reasons, Compont, an NBCA chemical adhesive, which is fast acting and provides good adhesive strength, is the preferred surgical adhesive used in China today [28]. The reason why additional suture fixation is needed is that the oesophageal hiatus is a very dynamic area with approximately 3,000 movements every day, therefore erosion, stricture or recurrence might happen with inadequate fixation [12]. However, without glue spray, the fixation needs more sutures, and therefore becomes more time consuming.

Proper mesh fixation is the key for the efficiency and the safety of hiatal hernia repair. Most surgeons currently use suture and some use tacks or staples for fixation. Both of these methods have their problems and do not allow strong, uniform, and immediate fixation of the mesh to the crural fibres. Having used the NCBA glue for mesh fixation in laparoscopic inguinal hernia repair since 2009 at our centre, we noticed that the fixation of the mesh was strong, immediate, and uniform [27]. On the other hand, the application of tacks at hiatus has been reported to cause serious complications such as cardiac tamponade and mortality [29]. As a result, tacker fixation has been strongly advised against by several surgeons [30]. In addition, the SAGES guidelines for management of hiatal hernia state that care should be taken about the mesh fixation technique. In particular, tacks can breach the aorta or pericardium when applied low on the left crus or anteriorly near the apex of the crura [30]. In comparison, medical glue fixation is safe without the risk of penetrating important organs. Furthermore, some studies reported that fixation by glue combined with suture is as strong as tacker [28].

In conclusion, biological mesh reinforcement of crural closure was safe and effective for repairing large hiatal hernias. Medical glue combined with suture can provide solid and secure fixation and can reduce the serious complications caused by fixation. The limitation of this study includes the small number of patients and relatively short follow up period. Ideally, a multiple center clinical study with control group could be carried out for further research.

## Conclusions

Biological mesh fixed with suture and medical glue was safe and effective for repairing large hiatal hernias. Of course, a longer follow-up is still needed for determining long-term outcomes.

## Data Availability

The datasets used and/or analysed during the current study are available from the corresponding author on reasonable request.

## Abbreviations

GERD: Gastroesophageal reflux disease
LHHR: Laparoscopic hiatal hernia repair
UGI: Upper gastrointestinal
NBCA: n-Butyl-2-cyanoacrylate
VAS: Visual analogue score
LESP: Lower esophageal sphincter pressure
SIS: Small intestinal submucosa
GI: Gastrointestinal
SAGES: Society of American Gsatrointestinal and Endoscopic Surgeons
